# Synergetic full-parametric Aharonov–Anandan and Pancharatnam–Berry phase for arbitrary polarization and wavefront control

**DOI:** 10.1515/nanoph-2025-0357

**Published:** 2025-10-16

**Authors:** Tong Liu, Yanzhao Wang, Weike Feng, Huiling Luo, ZhengJie Wang, Hui Wang, He-Xiu Xu, Xiangang Luo

**Affiliations:** Air and Missile Defense College, Air Force Engineering University, Xi’an 710051, China; State Key Laboratory of Optical Technologies on Nano-Fabrication and Micro-Engineering, Institute of Optics and Electronics, Chinese Academy of Sciences, Chengdu 610209, China

**Keywords:** full-parametric AA phase, chiral meta-atom, arbitrary polarization control

## Abstract

Electromagnetic devices with multiple polarization modes are urgently needed in remote sensing detection and radar imaging due to their ability to obtain scattering information from targets through manipulation of full-parametric Jones matrix components (*J*_*xx*_, *J*_*xy*_, *J*_*yx*_, *J*_*yy*_). Although metasurfaces exhibit exceptional capability for polarization control, they typically facilitate conversion between specific linearly polarized (LP) and circularly polarized waves on Poincaré sphere. Here, we find that identical phases in two co-polarized components is exactly half of the sum of phases in two cross-polarized components by deriving Jones matrix *J*_AAL_ with full-parametric Aharonov–Anandan (AA) phase Jones matrix. On this basis, a novel spin-decoupled paradigm is proposed by merging of AA phase and Pancharatnam–Berry (PB) phase mechanisms. Such a paradigm in diatomic metasurface is promised to achieve elegant amplitude-phase controlling and generate arbitrary polarized waves. For verification, two types of meta-devices were designed, fabricated, and experimentally characterized. Compared to the combination of propagation and PB phase, the proposed method enables simultaneous broadband arbitrary LP-to-LP conversion and wavefront control with a relative bandwidth of 43.5 %. Our strategy establishes theoretical foundation for spin-decoupled phase manipulation and amplitude-phase control of AA phase, providing a solid platform and guidance for the design of devices with arbitrary polarization and wavefront control.

## 1 Introduction

The ability to arbitrarily manipulate polarization states has become essential for applications such as remote sensing [1], [2], [3], [4], and imaging [5], [6], [7], enabling effective resolution of target signatures. Such a control fundamentally requires complete engineering of the full-parametric Jones matrix (*J*_*xx*_, *J*_*xy*_, *J*_*yx*_, *J*_*yy*_) to generate desired polarization response [8], [9], [10], [11]. Metasurfaces have emerged as integrated polarization control platforms [8], [9], [10], [12], [13], [14], [15], [16], [17], [18], [19], [20], [21], [22], [23], [24], achieving on-demand polarization tailoring through manipulation of two orthogonal Jones matrix components [25], [26], [27], [28], [29], [30] via diverse strategies including space-time coding [31], [32], [33], [34], multi-phase interference [23], [35], and lossy absorption [36]. Among these approaches, meta-atoms incorporating multi-phase mechanisms such as Pancharatnam–Berry (PB) phase, propagation phase, or detour phase [19], [37], [38] were not limited by spectral constraints and can be further extended to high frequencies [39], [40]. Researchers have attempted to employ multiple pure PB phase meta-atoms to form pixel for arbitrary polarization conversion and wavefront manipulation [41], [42]. However, PB phase emerges under CP wave illumination through rotating meta-atoms by *θ*, suffering from spin-locking constraints and yielding a phase difference of Δ*φ* = ±2*σθ* (where *σ* = +1/−1 corresponds to right/left-handed polarization, RCP/LCP) [43]. Therefore, PB phase-based metasurfaces produce conjugate images at symmetric mirrored positions [44]. In contrast, the propagation phase leverages dimension variations within LP basis [42], [44], [45], [46], [47], [48], and thus their intrinsic resonant dispersion effects severely restrict operational bandwidth.

To address spin-locking and bandwidth limitations, the Aharonov–Anandan (AA) phase in chiral meta-atoms achieves spin-decoupled functionality via non-adiabatic cyclic evolution of waves carrying spin angular momentum in meta-atoms. This geometric mechanism yields phase shifts governed by asymmetric arc length, achieving broadband operation (1.245 μm – 1.55 μm, 21.8 % fractional bandwidth) [49], [50], [51], [52], [53] via asymmetric evolution arcs by mimicking Aharonov–Bohm ring [54], [55]. However, most prior studies on chiral meta-atoms have focused primarily on the cross-circularly polarized Jones matrix components, resulting in limited polarization states (specific *x*/*y*-polarized or CP state) and thus making manipulation of AA phase particularly co-CP phase components ( $\left\langle \sigma_{ll}\right|$ and $\left\langle \sigma_{rr}\right|$ ) remains elusive. Furthermore, single AA phase strategy is unable to decouple phase and amplitude of two orthogonally polarized components. This poses challenges for metasurfaces to achieve arbitrary polarization operations.

**Figure 1:**
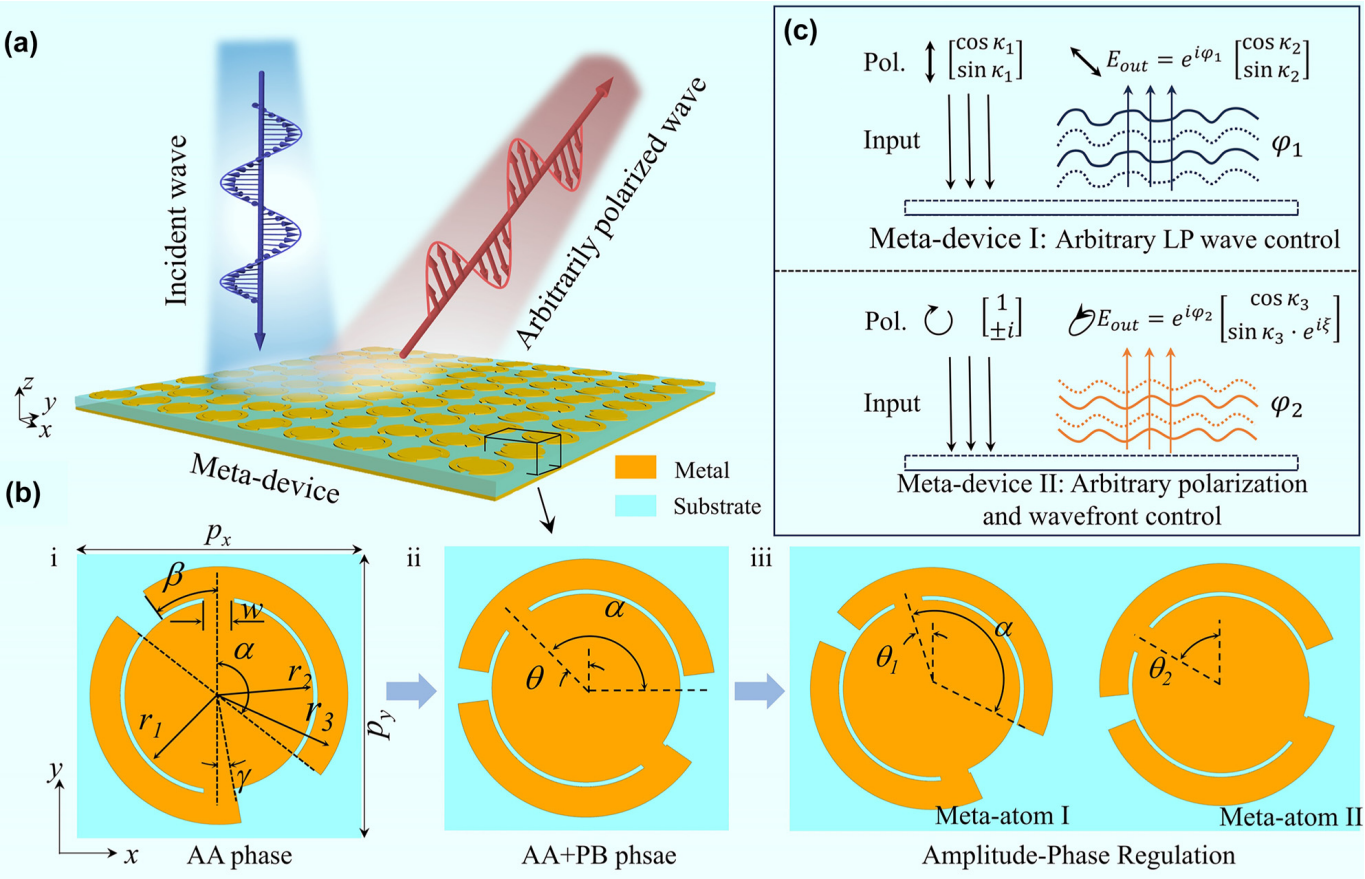
Schematic function of meta-devices based on AA phase. (a) Schematic function of AA phase polarization conversion meta-device. (b) Evolution of basic chiral meta-atom to spin-decoupled and diatomic spin-decoupled with amplitude-phase control based on AA phase. Detailed geometric parameters are *p*_*x*_ = *p*_*y*_ = 10 mm, *w* = 1 mm, *β* = 36°, *γ* = 8°, *r*_1_ = 3.1 mm, and *r*_2_ = 3.25 mm. Here, *w* denotes the width of connecting rod between metallic arc and inner disk, *p*_*x*_ and *p*_*y*_ represent the periodicity of meta-atom in *x*- and *y*-directions, respectively, and *θ*, *θ*_1_, and *θ*_2_ denote orientation angles of the top metal patch of meta-atoms. (c) Schematic function of two types of arbitrary polarization conversion and wavefront control.

Here, we report a novel strategy for generating arbitrary polarization state by combining full-parametric AA phase of chiral meta-atoms and PB phase. The contribution of our concept to EM wave manipulation is that a general amplitude-phase relationship for each scattering component of Jones matrix of AA phase is derived based on unitarity, symmetry, and lossless features, and then the extended Jones matrix with AA phase is further developed for broadband amplitude-phase controlling. The importance and significance of our work can be inspected from Figure 1a, which shows that the input LP or CP wave on a meta-device with our proposed integrated AA and PB phases can be converted into output wave with arbitrary polarization state and phase. For verification and potential applications, two types of proof-of-concept meta-devices have been designed to achieve arbitrary polarization. The first meta-device converts *y*-polarized wave into ±45° LP waves and focuses their energy to different positions, while the second one transfers CP wave into arbitrarily polarized state with elegant ability of wavefront control. Our approach not only opens an alternative avenue for conversion between arbitrary polarization states and complicated wavefront shaping, but also provides a general approach to analyzing the complete EM properties of metasurface platforms. This method holds promise for applications in integrated systems, such as polarization multiplexing, polarization detection and communication, etc.

## 2 Results and discussions

Theoretically, an arbitrarily polarized wave can be decomposed into two orthogonal CP components with distinct amplitude and phase response. To synthesize arbitrary states of polarization, we designed a chiral meta-atom leveraging AA phase for spin-dependent phase control, enabling independent manipulation of two cross-CP components, as seen in inset (i) of Figure 1b. The reflective meta-atom comprises a 2-mm-thick F4B substrate sandwiched by top metal patch and bottom metal ground. To increase cross-polarized scattering coefficients across a wide bandwidth, the top patch is designed consisting of a centered metallic disk and dual identical arc-shaped metallic patterns attached to the disc with rectangular patches. Parameters *β* and *γ* were meticulously optimized through full-wave electromagnetic simulations and subsequently fixed at 36° and 8°, respectively. The arc angle *α* of the left upper and right bottom arm is dynamically adjusted in accordance with different phase requirements.

For arbitrary LP-to-LP wave conversion, AA phase and PB phase are synergistically integrated to decouple EM waves of two cross-CP components ( $\left\langle \sigma_{lr}\right|$ and $\left\langle \sigma_{rl}\right|$ ) by simultaneously changing parameter *α* and orientation angle *θ* of the top patch. This meta-atom is referred to as spin-decoupled meta-atom (inset (ii) of Figure 1b). Hence, the incident arbitrary LP state $\left[\begin{array}{c} \cos\kappa_{1}\\ \sin\kappa_{1} \end{array}\right]$ can be transformed into arbitrary output LP state $\left[\begin{array}{c} \cos\kappa_{2}\\ \sin\kappa_{2} \end{array}\right]$ with independent phase profile *ϕ*_1_ based on spin-decoupled strategy (top panel of Figure 1c). For the conversion from CP wave to arbitrary polarized wave, amplitude and phase responses of $\left\langle \sigma_{rl}\right|$ ( $\left\langle \sigma_{lr}\right|$ ) and $\left\langle \sigma_{ll}\right|$ ( $\left\langle \sigma_{rr}\right|$ ) components can be manipulated based on our full-parametric AA and PB phase Jones matrix framework. This control is implemented through diatomic meta-atoms consisting of a pair of spin-decoupled meta-atoms with different *θ*, as shown in (iii) of Figure 1b. As a result, the incident CP $\left[\begin{array}{c} 1\\ \pm i \end{array}\right]$ can be transformed into arbitrary output polarization state $\left[\begin{array}{c} \cos\kappa_{3}\\ \sin\kappa_{3}{\text{e}}^{\text{i}\xi } \end{array}\right]$ with independent phase profile *ϕ*_2_, as illustrated in bottom panel of Figure 1c. Here, *κ*_1_ and *κ*_2_ (*κ*_3_) denote polarization angle of input and output LP (arbitrarily polarized) waves, respectively, and *ξ* represents the phase difference between two output orthogonal polarized components.

### 2.1 Concept and fundamentals

#### 2.1.1 AA phase mechanism

The realization of arbitrary polarization conversion through Aharonov–Anandan (AA) phase demands precise control over both amplitude and phase responses of each Jones matrix element. While previous chiral metamaterials have achieved spin-dependent phase manipulation, our framework advances this paradigm by analyzing the relationships within the Jones matrix components of chiral meta-atom and achieving elegant amplitude-phase control. Firstly, we analyze a reflective meta-atom’s EM properties through the linear Jones matrix $J_{\text{lin}}=\left[\begin{array}{cc} \left|r_{xx}\right|{\text{e}}^{xx} & \left|r_{xy}\right|{\text{e}}^{xy}\\ \left|r_{yx}\right|{\text{e}}^{yx} & \left|r_{yy}\right|{\text{e}}^{yy} \end{array}\right]$ in Cartesian base. Here, |*r*_*xy*_| (*φ*_*xy*_) and |*r*_*yy*_| (*φ*_*yy*_) represent reflection amplitude (phase) responses of *x*- and *y*-polarized components under excitation of *y*-polarized wave (the same nomenclature to other parameters). Then, the *J*_lin_ can be transformed into circular base as *J*_cir_ (*J*_cir_ = Λ^−1^ *J*_lin_Λ) through transformation factor $\Lambda =\frac{1}{\sqrt{2}}\left[\begin{array}{cc} 1 & 1\\ i & -i \end{array}\right]$ , and simplified according to symmetry [56], lossless and unitarity [46], [57] features (see more details in Supporting Information Section S1)

(1) $$\begin{array}{cc} J_{\text{cir}} & =\left[\begin{array}{cc} \left|r_{ll}\right|{\text{e}}^{\varphi_{ll}} & \left|r_{lr}\right|{\text{e}}^{\varphi_{lr}}\\ \left|r_{rl}\right|{\text{e}}^{\varphi_{rl}} & \left|r_{rr}\right|{\text{e}}^{\varphi_{rr}} \end{array}\right]\\ & =\left[\begin{array}{cc} \left|r_{xx}\right|\cos\left(\Delta \right)\cdot {\text{e}}^{\text{i}\Phi } & \sqrt{1-{\left(\left|r_{xx}\right|\cos\left(\Delta \right)\right)}^{2}}{\text{e}}^{\text{i}\arctan\left(\frac{\mp \left|r_{yx}\right|}{\left|r_{xx}\right|\sin\left(\Delta \right)}\right)}{\text{e}}^{\text{i}\Phi }\\ \sqrt{1-{\left(\left|r_{xx}\right|\cos\left(\Delta \right)\right)}^{2}}{\text{e}}^{\text{i}\arctan\left(\frac{\pm \left|r_{yx}\right|}{\left|r_{xx}\right|\sin\left(\Delta \right)}\right)}{\text{e}}^{\text{i}\Phi } & \left|r_{xx}\right|\cos\left(\Delta \right)\cdot {\text{e}}^{\text{i}\Phi } \end{array}\right] \end{array}$$

where $\Delta =\left(\varphi_{xx}-\varphi_{yy}\right)/2$ , and $\Phi =\left(\varphi_{xx}+\varphi_{yy}\right)/2$ . This analysis yields a universal conclusion: the sum of phases in co-polarized components equals that of two cross-polarized scattering components ( $\varphi_{ll}=\varphi_{rr}=\frac{\varphi_{rl}+\varphi_{lr}}{2}$ ), which is applicable to systems with symmetry, unitarity, and losslessness. Here, we investigate the scenario characterized by phase *φ*_*rl*_ = *φ*_AA_ and *φ*_*lr*_ = 0, where *φ*_AA_ represents AA phase in $\left\langle \sigma_{rl}\right|$ induced by changing arc angle *α* of the arm. Finally, Jones matrix *J*_AAL_ with AA phase of meta-atom is presented as below:

(2) $$J_{\text{AAL}}=\left[\begin{array}{cc} \left|r_{ll}\right|{\text{e}}^{\text{i}\frac{\varphi_{\text{AA}}}{2}} & \sqrt{1-{\left|r_{rr}\right|}^{2}}\\ \sqrt{1-{\left|r_{ll}\right|}^{2}}{\text{e}}^{\text{i}\varphi_{\text{AA}}} & \left|r_{rr}\right|{\text{e}}^{\text{i}\frac{\varphi_{\text{AA}}}{2}} \end{array}\right]$$

**Figure 2:**
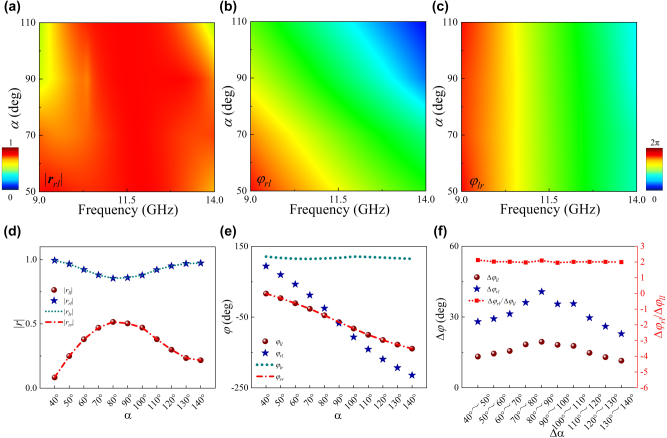
EM characterization of proposed chiral meta-atom based AA phase mechanism. Numerically calculated reflection magnitude and phase response at 9–14 GHz for (a–b) $\left\langle \sigma_{rl}\right|$ and (c) $\left\langle \sigma_{lr}\right|$ under LCP(RCP) wave illumination. (d) The amplitude and (e) phase responses across co-polarized ( $\left\langle \sigma_{ll}\right|/\left\langle \sigma_{rr}\right|$ ) and cross-polarized ( $\left\langle \sigma_{lr}\right|/\left\langle \sigma_{rl}\right|$ ) component at 10 GHz. (f) The phase shifts and its ratio between co- and cross-polarized components (Δ*φ*_*ll*_ and Δ*φ*_*rl*_) by tuning *α* from 40° to 140° at 10° intervals under LCP wave excitation at 10 GHz.

A basic chiral meta-atom prototype was designed and numerically characterized to validate above theory, as shown in inset (i) of Figure 1b. Figure 2 and Table S1 presents the reflected amplitude and phase response of the proposed meta-atom under normal LCP wave illumination, demonstrating |*r*_*rl*_| > 0.75 across 9–14 GHz while satisfying the lossless condition (|*r*_*ll*_|^2^ + |*r*_*rl*_|^2^ ≈ 1). Similar behavior is observed under RCP wave excitation (Figure 2d), where |*r*_*lr*_| = |*r*_*rl*_| and |*r*_*rr*_|^2^ + |*r*_*lr*_|^2^ ≈ 1. A larger phase cover of 270° with quasi-nondispersive properties is achieved in $\left\langle \sigma_{rl}\right|$ ( $\varphi_{rl}=\varphi_{\text{AA}}\approx 2\int \text{d}\left(\Delta \alpha \right)$ ) by tuning *α* with *φ*_*lr*_ fixed to zero (Figure 2b, c, and e), indicating that the meta-atom exhibits broadband spin-dependent phase shift across 9–14 GHz. Although the AA phase is intrinsically wavelength-independent, the meta-atoms required for parameter control may introduce undesired material dispersion and structural resonances which would limit the operational bandwidth. Moreover the theoretical calculations indicate that phase variation in $\left\langle \sigma_{ll}\right|$ is half of those in $\left\langle \sigma_{rl}\right|$ (Figure 2f), and this conclusion also applies to phase of $\left\langle \sigma_{rr}\right|$ . These results are in good agreement with those calculated by theoretical derivation in Eq. (2), validating our proposed theory of AA phase. Additionally, the proposed methodology can be universally extended to achieve simultaneous phase shifts in both $\left\langle \sigma_{lr}\right|$ and $\left\langle \sigma_{rl}\right|$ through spin-dependent geometric paths engineering of meta-atom, as theoretically derived in Eqs. (S5) and (S6) (Supporting Information).

#### 2.1.2 Jones matrix of phase and amplitude-phase control based on synergetic AA and PB phase mechanisms

To independently adjust the phase *φ*_*ll*_, *φ*_*lr*_, and *φ*_*rl*_, the methods of spin-decoupled phase and amplitude-phase manipulation are proposed by simultaneously manipulating *α* and *θ* of meta-atoms. For amplitude-phase manipulation, a diatomic meta-atom is designed which consists of two spin-decoupled chiral meta-atoms (meta-atom I and II) with the same *α* but different *θ*, as illustrated in inset (iii) of Figure 1b. Cross- and co-polarized scattering coefficients are controlled by the angle difference between two meta-atoms under excitation of CP wave based on far-field interference, as shown in ${\text{e}}^{\text{i}2\theta_{1}}+{\text{e}}^{\text{i}2\theta_{2}}=2\text{cos}\left(\theta_{2}-\theta_{1}\right)\cdot {\text{e}}^{\text{i}\cdot \left(\theta_{1}+\theta_{2}\right)}$ . Consequently, the Jones matrix for diatomic meta-atom can be expressed as follows:

(3) $$J_{\text{PB }+\text{ AAL}}=\left[\begin{array}{cc} \sqrt{1-{\left(\left|r_{rl}\right|\cos\left(\theta_{2}-\theta_{1}\right)\right)}^{2}}{\text{e}}^{\text{i}\frac{\varphi_{\text{AA}}}{2}} & \left|r_{rl}\right|\cos\left(\theta_{2}-\theta_{1}\right){\text{e}}^{\text{i}\left(\theta_{2}+\theta_{1}\right)}\\ \left|r_{rl}\right|\cos\left(\theta_{2}-\theta_{1}\right){\text{e}}^{\text{i}\left(\varphi_{\text{AA}}-\theta_{1}-\theta_{2}\right)} & \sqrt{1-{\left(\left|r_{rl}\right|\cos\left(\theta_{2}-\theta_{1}\right)\right)}^{2}}{\text{e}}^{\text{i}\frac{\varphi_{\text{AA}}}{2}} \end{array}\right]$$

For verification, reflection amplitude and phase response of diatomic meta-atom as orientation angle difference Δ*θ* (Δ*θ* = *θ*_2_ − *θ*_1_, *θ*_1_ = 0) varying from 0° to 90° were numerically simulated within 9–13 GHz, as illustrated in Figure S7 (Supporting Information). The results demonstrate that |*r*_*rl*_| and |*r*_*lr*_| decrease from near unity to 0.24 (−12.4 dB) as Δ*θ* increases within a wide frequency band 9.8–11.8 GHz, while the trend is opposite for |*r*_*ll*_| (Figure S7a and b). The numerically calculated reflection intensity |*r*_*rl*_| are consistent with counterparts of theoretically calculated by Eq. (3), as shown in Figure S7c. It is evident that phase shifts occurs in both $\left\langle \sigma_{rl}\right|$ and $\left\langle \sigma_{lr}\right|$ as Δ*θ* increase, while phase of $\left\langle \sigma_{ll}\right|$ and $\left\langle \sigma_{rr}\right|$ remain nearly unchanged (Figure S7d and f in Supporting Information).

### 2.2 Arbitrary LP wave control using spin-decoupled phase manipulation

**Figure 3:**
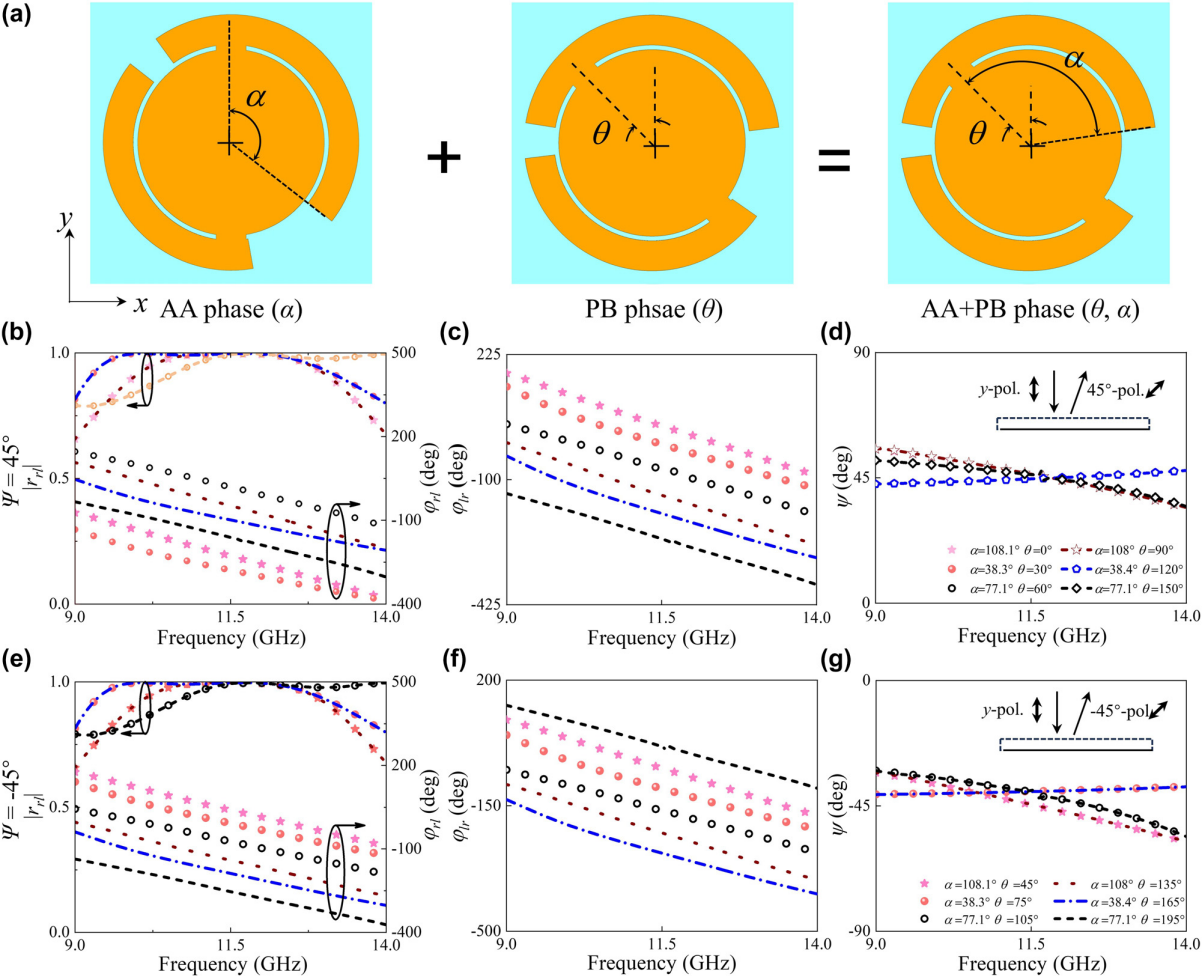
Structure and EM response of spin-decoupled meta-atoms with synergetic AA and PB phase mechanisms. (a) Structural evolution of meta-atoms implementing from geometric phase to synergetic AA and PB phases, where AA phase is tuned via arc angle *α* while PB phase is controlled through orientation angle *θ*, respectively. Numerically calculated amplitude (|*r*_*rl*_| and |*r*_*lr*_|) and phase response (*φ*_*rl*_ and *φ*_*lr*_) of cross-circular polarization components for meta-atoms A (b–c) and B (e–f) under LCP and RCP wave illumination, where meta-atoms A and B represent structures transforming *y*-polarized wave to 45° and −45°-LP waves, respectively. Polarization angle of meta-atoms for *y*-polarized wave to (d) 45° and (g) −45° LP waves, where *ψ* = (*φ*_*rl*_ *– φ*_*lr*_)/2.

**Figure 4:**
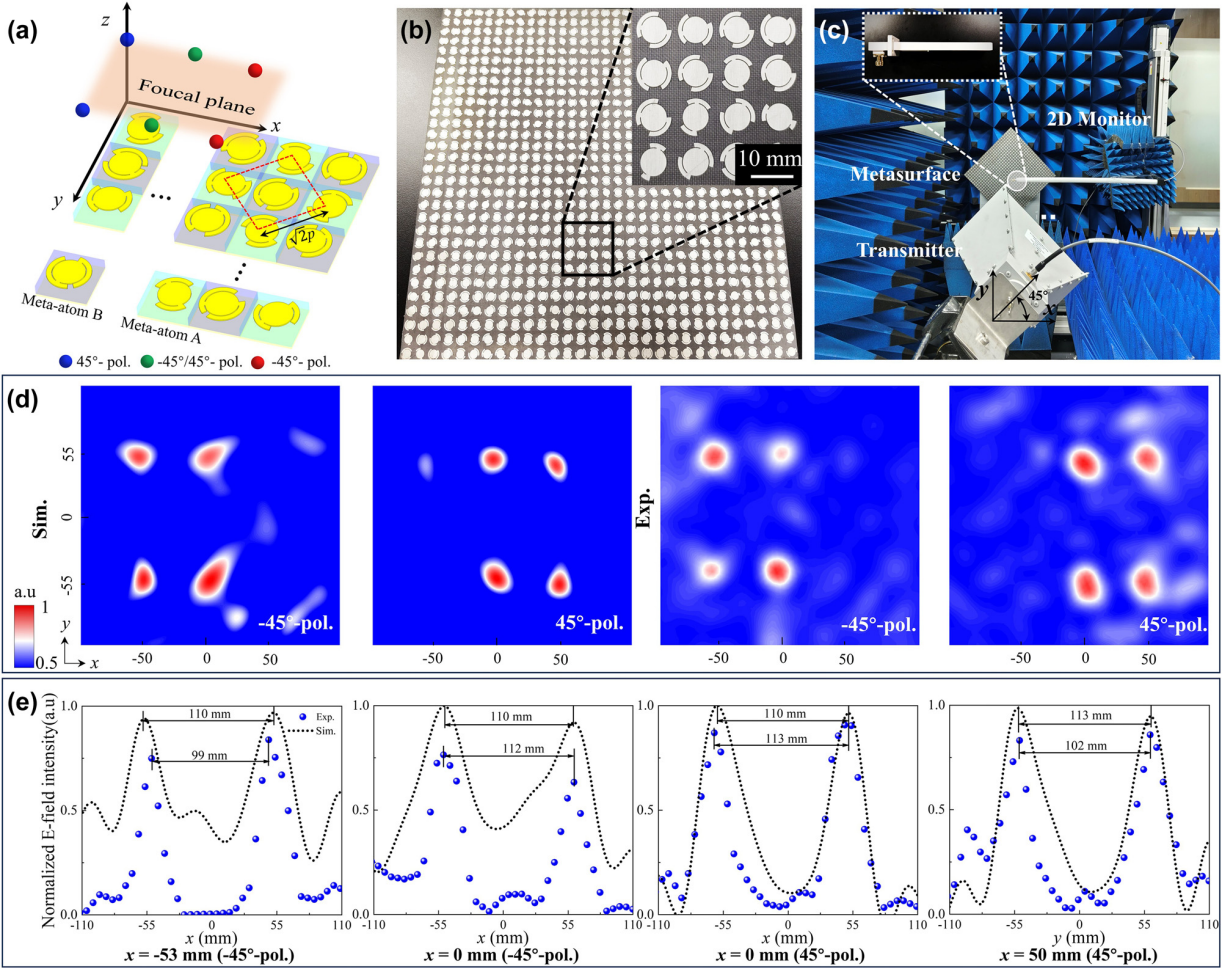
Experimental characterization of the spin-decoupled planar multifocal lens for arbitrary LP states. (a) Meta-device array layout, where dashed line represents real periodicity *p*_*r*_ of meta-atoms A and B. (b) Photograph of the fabricated sample of planar multifocal lens. (c) Near-field experimental setup. (d–e) Simulated and measured normalized 2D electric field intensity |*E*_45°_| and |*E*_−45°_| at 11.1 GHz in *xoy* plane (*z* = 204 mm), with cross-sectional profiles along *y* at *x* = −53, 0, 0, and 50 mm.

To achieve arbitrary LP-to-LP conversion and wavefront control, we implement spin-decoupled phase manipulation via meta-atoms (Figure 3a) featuring simultaneous modulation of arc angle *α* for AA phase control and orientation angle *θ* for PB phase engineering. As indicated from Eq. (S7), this equation can be further simplified as $J_{\text{PB }+\text{ AAL}}=\left[\begin{array}{cc} 0 & {\text{e}}^{\text{i}2\theta }\\ {\text{e}}^{\text{i}\left(\varphi_{\text{AA}}-2\theta \right)} & 0 \end{array}\right]$ with *φ*_*rl*_ = *φ*_AA_ − 2*θ* and *φ*_*lr*_ = 2*θ* when |*r*_*lr*_| = |*r*_*rl*_| = 1, which evidently demonstrates that phases of *φ*_*rl*_ and *φ*_*lr*_ are spin decoupled. The polarization angle of arbitrary LP-to-LP conversion is governed by *ψ* = (*φ*_*rl*_ – *φ*_*lr*_)/2 = *φ*_AA_/2−2*θ*, while the associated phase retardation (Δ*φ* = *φ*_AA_/2) dictates the polarization-dependent wavefront shaping capability. Figure 3b–e present simulated amplitude (|*r*_*rl*_| and |*r*_*lr*_|) and phase (*φ*_*rl*_ and *φ*_*lr*_) responses of two types of LP conversion meta-atoms libraries (meta-atoms A and B) under LCP or RCP wave illumination, satisfying the spin-decoupled condition of |*r*_*lr*_| = |*r*_*rl*_| ≈ 1 and full 2*π* phase cover with quasi-nondispersive performance across 9–14 GHz. The polarization angle of reflected wave approximates 45° and −45° under excitations of *y*-LP wave (Figure 3d and g), respectively. To verify the proposed method, a focusing polarization conversion metasurface was designed using meta-atoms established in Figure 3. As demonstrated in Supplementary Figure S6, high-quality focusing is achieved across 9–14 GHz (43.5 % fractional bandwidth), confirming the non-dispersive behavior in metasurface with synergetic AA and PB phase mechanisms. Furthermore, multi-focus planar lens integrating two types of distinct meta-atoms A and B with polarization and phase control capabilities was designed via complex-phase addition theorem [58] and diagonally interleaved method. Orthogonal output polarized states between meta-atoms A and B ensures negligible polarized crosstalk. To enhance resolution, an interleaved coding was employed where each pair of meta-atoms (A and B) with periodicity *p*_*r*_ ( $p_{r}=\sqrt{2}p$ ) forms a minimal functional unit, see Figure 4a. The corresponding EM response (amplitude, phase, polarization) of meta-atoms and phase distributions are detailed in Figure S11 (Supporting Information). The phase *φ* ^*mn*^ for each meta-atom of multi-focus planar lens meta-device can be calculated through complex-phase superposition formula ( $\varphi^{mn}=\arg\left(\sum_{i=1}^{4}{\text{e}}^{-j\varphi_{i}^{mn}}\right)$ ), where $\varphi_{i}^{mn}$ denotes the phase of the *mn*-th meta-atom required to generate *i*-th focusing beam. Ultimately, *y*-polarized waves are converted into ±45° LP states after impinging the meta-device and focused on distinct spatial positions (Figure 4a).

For experimental demonstration, a porotype of planar multifocal lens was fabricated with sample shown in Figure 4b and characterized using the near-field experimental setup illustrated in Figures 4c and S12a, where LP horn antenna and sample were turned around 45° clockwise. Two horizontally and vertically oriented waveguide probes were employed to capture reflected static EM signals from −45° and 45° LP waves, respectively. Upon interaction with meta-atom A, the *y*-polarized incident wave was transformed into a −45° LP wave, generating four localized energy foci at coordinates (−53, −49.5 mm), (−53, 49.5 mm), (0, −56 mm), and (0, 56 mm) within *xoy* plane (*z* = 204 mm). These measured foci characterized by 0.6 power beam width of field pattern normalized to the maximized intensity exhibited a size of 8.3, 19.3, 19.4 and 2.8 mm, respectively. Similarly, meta-atom B achieved conversion of *y*-polarized waves to 45° LP state with energy foci at (0, −55.5 mm), (0, 55.5 mm), (50, −56.5 mm), and (50, 56.5 mm) in the same plane. The corresponding focal sizes are 16.6 mm, 24.9 mm, 11.1 mm, and 19.3 mm. Simulated and experimental focal positions at 11.1 GHz align closely with theoretical predictions, as demonstrated in Figure 4d and e. Furthermore, two-dimensional (2D) electric field distributions in *yoz* planes at *x* = −53 mm, 0 mm, and 50 mm (Figures S9 and S10) clearly illustrate the designed multi-focus behavior with high spatial resolution and cross-polarization suppression across 10–12 GHz. The discrepancy between the simulated and measured multiple foci observed in Figure 4e may have resulted from differences in the selection of the focal plane (Figures S9 and S10). Relatively narrower bandwidths between meta-atoms in Figure 3 and array originate primarily from phase errors induced by near-field coupling and resonant frequency shift of meta-atoms as the periodicity increases from *p* to *p*_*r*_ (Figures S11 and S12). Additionally, laterally distributed multifoci inherently induce chromatic dispersion under broadband operation, resulting in undesired focal elongation along *x* axis. The measured focusing efficiency, defined as the ratio between the powers carried by the focal spot of 45° (−45°) LP and the incident beam, was calculated as 91 % (89 %). Large signal intensity is received based on simultaneous matching of polarization states and spatial position, confirming the meta-device’s capability for arbitrary LP-to-LP conversion and high-precision wavefront control.

### 2.3 Arbitrary polarization and wavefront control using amplitude-phase control

**Figure 5:**
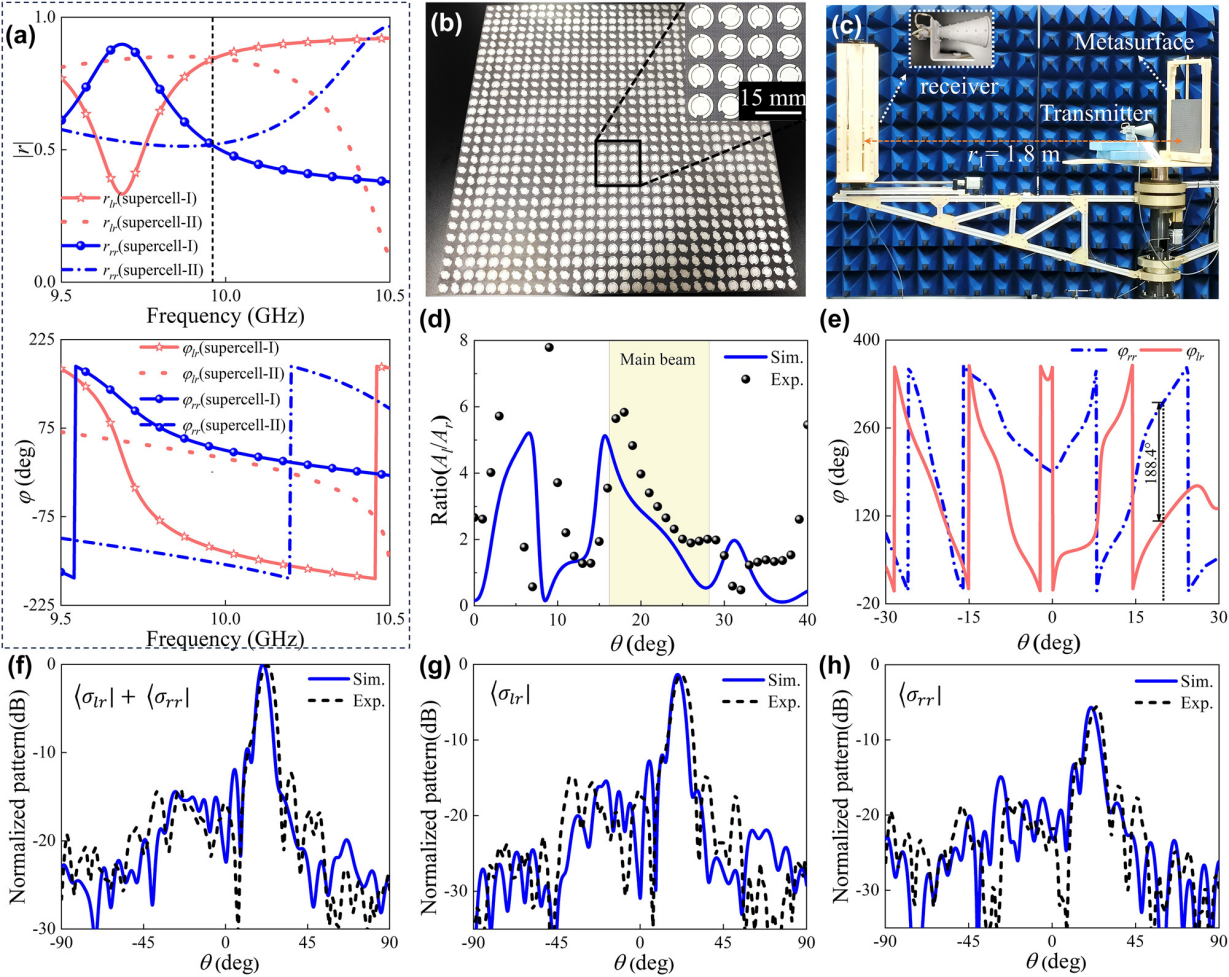
Experimental characterization of diatomic meta-device with amplitude-phase control for arbitrary polarization and wavefront manipulation. (a) Simulated amplitude (|*r*_*lr*_| and |*r*_*rr*_|) and phase (|*φ*_*lr*_| and |*φ*_*rr*_|) responses of the 1-bit meta-atoms under RCP wave illumination. (b) Photograph of the fabricated sample. (c) Far-field experimental setup. (d) Amplitude ratio and (e) phase difference between LCP and RCP components. (f–h) Simulated and experimental 1D far-field scattering patterns at *f* = 10 GHz in *xoz* plane. Here (f), (g), and (h) correspond to that of total, LCP, and RCP components of scattered wave.

To enable arbitrary polarization detection and communication, the polarization state of incident EM wave needs to be converted into arbitrary states of polarization. Here, we demonstrated the conversion of CP states to arbitrary states of polarization alongside wavefront control via the amplitude-phase manipulation. As shown in Eq. (S4), the co-polarized phase response follows *φ*_*rr*_ = *φ*_AA_/2, while the cross-polarized phase shifts are governed by *θ*_1_ + *θ*_2_ under RCP wave illumination. This configuration enables precise polarization engineering through the derived polarization angle *ψ* = *φ*_AA_/4 − (*θ*_1_ + *θ*_2_)/2 and phase retardation Δ*φ* = *φ*_AA_/4 + (*θ*_1_ + *θ*_2_)/2. The ellipticity manipulation of the anisotropic diatomic meta-atom is dictated by the orientation angle difference Δ*θ* = *θ*_2_ − *θ*_1_, establishing a mapping between geometric parameter space and full-Stokes polarization control. Furthermore, we validate the applicability of this strategy through the design and characterization of functional meta-device. According to the relationship between arbitrary polarization parameters and components of CP wave ( $\chi =\frac{1}{2}\text{si}{\text{n}}^{-1}\frac{A_{r}^{2}-A_{l}^{2}}{A_{r}^{2}+A_{l}^{2}}$ and $\Psi =\frac{\varphi_{r}-\varphi_{l}}{2}$ ) [44], the elliptical angle *χ* and polarization angle *ψ* of EM wave can be collaboratively manipulating the amplitude ratio *A*_*l*_/*A*_*r*_ and phase difference of two orthogonal CP components, where *A*_*r*_ and *A*_*l*_ denote reflection magnitude for $\left\langle \sigma_{lr}\right|$ and $\left\langle \sigma_{rr}\right|$ , respectively. Although phase cover of $\left\langle \sigma_{rl}\right|$ can be extend to 2*π* through altering *α*, phase of $\left\langle \sigma_{ll}\right|$ is only half of that in $\left\langle \sigma_{rl}\right|$ according to Eq. (S4) in Supporting Information. Theoretically, the additional *π* phase jump in $\left\langle \sigma_{ll}\right|$ or $\left\langle \sigma_{rr}\right|$ can be continued by further merging the AA phase in $\left\langle \sigma_{lr}\right|$ component, as illustrated in Eq. (S6) (Supporting Information). Here, 1-bit coding diatomic meta-atoms presented in inset (iii) of Figure 1b were adopted to design meta-device that convert RCP wave into left-handed elliptically polarized wave (*ψ* = 90°, *χ* = 13.5°), while simultaneously satisfying both the phase cover and the amplitude ratio *A*_*l*_/*A*_*r*_ at 9.96 GHz (Figure 5a). The relatively narrower bandwidth observed in Figure 5a compared to the meta-atoms in Figure 3 results from multi-resonance behavior, and spectral shifts in both co- and cross-polarized components induced by parameter adjustments. The detailed geometric parameters of diatomic meta-atoms are *α* = 46.6°, *θ*_1_ = −52.1°, and *θ*_2_ = −16.8° for *φ* = 0, and *α* = 137.8°, *θ*_1_ = 17.1°, and *θ*_2_ = 59.8° for *φ* = *π*. Simulations reveal that both types of meta-atoms exhibit minor amplitude and phase variations under 30° oblique incidence (Figure S13, more details in Supporting Information Section S5), where edge-positioned meta-atoms of the array exerting negligible influence on the scattered wave’s polarization state.

For proof-of-principle verification, a 15 × 15 beam-steering diatomic meta-device was designed with linear phase distribution shown in Figure S14. The porotype was fabricated with sample shown in Figure 5b and measured using the far-field experimental setup shown in Figures 5c and S10b. For quantitative and intuitive characterization, the linear amplitude ratio and phase difference of $\left\langle \sigma_{rr}\right|$ and $\left\langle \sigma_{lr}\right|$ under RCP wave excitation are firstly calculated, see Figure 5d and e. Numerical and experimental elliptical angles of main beam coincide well and are observed as 13.6° and 13.4° at 10 GHz for total component, respectively. Since background scattering in nonideal measurement setup inherently perturbs the radiation pattern of meta-device II, the sidelobes below −12.3 dB exhibit high sensitivity to scattering. However, this nonideal scattering pose has little effect to the main beam (exhibits robustness to such perturbations) due to concentrated energy of main beam. This sharp contrast accounts for large and minor deviations between simulated and measured LCP/RCP component ratio observed in sidelobe at *θ* = 30° and main beam. Moreover, the simulated polarization angle of 94.2° $\left(\Psi =\frac{\varphi_{rl}-\varphi_{ll}}{2}\right)$ is very close to that (90°) of target polarization state, indicating that incident CP wave is successfully converted into left-handed elliptically polarized wave. Figure 5f–h compare simulated and measured far-field scattering patterns whose deflection angles of main beam are observed as 20.5° and 23°, respectively which are close to the predicted one of 20°. The LCP and RCP components exhibit different scattered intensity, and they combine in space to form an elliptical polarization state. The measured half-power beamwidth of main beam was 8.1°, and the sidelobe level was −12.3 dB. The anomalous deflection efficiency, defined as ratio between the power of the main beam and totally reflected power, is calculated as 83.1 %. To further validate the control relationship for co-polarized component derived in Eq. (S8), we designed an additional polarization-preserving metasurface simultaneously maintained incident polarization and imparted desired wavefront phases, validating the amplitude-phase control strategy (Figure S15). To sum up, the above results demonstrate that the diatomic meta-device can effectively convert RCP wave into left-handed elliptical CP wave and thus validate the feasibility of arbitrary polarization control based on our proposed AA phase.

## 3 Conclusions

In summary, we have proposed a phase-amplitude manipulation strategy by synergistically combining full-parametric AA phase and PB phase. This approach is established based on unitary, symmetric, and lossless features of the Jones matrix, and validated through a meta-atom platform that simultaneously achieves arbitrary LP-to-LP conversion and wavefront manipulation across 9–14 GHz with 43.5 % fractional bandwidth. Two proof-of-concept meta-devices were designed: A planar multifocal lens converting *y*-polarized wave to ±45° LP waves and generating four predefined foci with >89 % efficiency; A diatomic meta-device transforming RCP wave into left-handed elliptically polarized wave with beam steering, exhibiting 83.1 % anomalous reflection efficiency. Both numerical simulations and experimental measurements confirm the effectiveness of our strategy. More importantly, theoretical derivation establishes a foundation for advanced applications of AA phase in arbitrary polarization synthesis.

## 4 Experimental section

For verification, the meta-devices were designed, numerically characterized and experimentally fabricated and measured. Both meta-devices (30 × 30 array, 300 × 300 mm^2^) were designed using CST Microwave Studio with open boundary conditions in *xyz* directions. The prototypes were fabricated via standard PCB processing, and the near- and far-field EM performances were measured by an AV3672B vector network analyzer (Figure S16 in Supporting Information). For near-field measurements, an LP horn antenna was positioned 1.6 m from the meta-device to ensure planar wavefront illumination. A 2D motor-mounted waveguide probe scanned a 350 × 350 mm^2^ area to map field intensities. For far-field characterization, an RCP transmitter was fixed at *z* = 205 mm (focal-diameter ratio *F*/*D* = 0.7) and dual LCP/RCP receivers were placed 1.8 m away, sweeping an angle from 0° to 180°.

## Supplementary Material

Supplementary Material Details
