# Supplementary material for: Synergetic full-parametric Aharonov–Anandan and Pancharatnam–Berry phase for arbitrary polarization and wavefront control
Source: Nanophotonics. 2025 Oct 16;14(22):3657–67. doi: 10.1515/nanoph-2025-0357 (PMC12592980; doi:10.1515/nanoph-2025-0357)
Supplement: Supplementary file 1 — Supplementary Material Details [file j_nanoph-2025-0357_suppl_001.docx]

# Supporting Information

**Synergetic full-parametric Aharonov-Anandan and Pancharatnam-Berry phase for arbitrary polarization and wavefront control**

Tong Liu,^a^ Yanzhao Wang,^a^ Weike Feng,^a^ Huiling Luo,^a^ ZhengJie Wang,^a^ Hui Wang,^a^ Xiangang Luo,^b,*^ He-Xiu Xu^a,*^

^a^ Air and Missile Defense College, Air force Engineering University, Xi'an 710051 (China)

^b^ State Key Laboratory of Optical Technologies on Nano-Fabrication and Micro-Engineering, Institute of Optics and Electronics, Chinese Academy of Sciences, Chengdu 610209 (China)

E-mail: [hxxuellen@gmail.com](mailto:hxxuellen@gmail.com) (He-Xiu Xu), [lxg@ioe.ac.cn](mailto:lxg@ioe.ac.cn) (Xiangang Luo)

**Keywords:** Full-parametric AA phase, chiral meta-atom, arbitrary polarization control

1. Derivation and expansion of Jones matrix with full-parameter AA phase
2. Jones matrix for AA and PB spin-decoupled metasurface
3. Jones matrix and EM response for diatomic spin-decoupled metasurface with amplitude-phase controlling
4. Simulated model and results of multifocal meta-device
5. Simulated model and experimental setups of diatomic meta-device with beam deflection

## **Section S1. Derivation and expansion of Jones matrix with full-parameter AA phase**

To obtain the Jones matrix with full-parameter AA phase, electromagnetic (EM) response of all components of the Jones matrix need be analyzed. Initially, the Jones matrix *J*_lin_ in Cartesian base was converted into circular base (*J*_cir_) through transformation factor $\text{Λ}=\frac{1}{\sqrt{2}}\left[\begin{matrix} 1 & 1 \\ i & -i \end{matrix}\right]$. The corresponding Jones matrix form in circular base is given as

$\begin{matrix} J_{\text{cir}}=\Lambda^{-1}J_{\text{lin}}\Lambda\\ =\frac{1}{2}\left[\begin{matrix} 1 & -i \\ 1 & i \end{matrix}\right]\left[\begin{matrix} r_{xx} & r_{xy} \\ r_{yx} & r_{yy} \end{matrix}\right]\left[\begin{matrix} 1 & 1 \\ i & -i \end{matrix}\right] \\ =\left[\begin{matrix} |r_{xx}|\cos(\Delta)\cdot e^{i\Phi} & \left(|r_{xx}|\sin(\Delta)e^{i\cdot\frac{-\pi}{2}}-i|r_{xy}|e^{i\frac{\pm\pi}{2}}\right)e^{i\Phi} \\ \left(|r_{xx}|\sin(\Delta)e^{i\cdot\frac{-\pi}{2}}+i|r_{xy}|e^{i\frac{\pm\pi}{2}}\right)e^{i\Phi} & |r_{xx}|\cos(\Delta)\cdot e^{i\Phi} \end{matrix}\right] \end{matrix}$ (S1)

where , and . The proposed meta-atom does not incur additional energy loss during modulating electromagnetic waves, indicating that the unitarity condition is satisfied. The energy of the incident wave equals the sum of the energies of transmitted and reflected waves. And the phase relationship between different components of the Jones matrix is. Therefore, the Jones matrix *J*_cir_ in Eq. S1 can be further simplified based on symmetry and unitarity condition as

$J_{_{\text{cir}}}^{'}=\left[\begin{matrix} |r_{xx}|\text{cos}\left(\Delta\right)\cdot e^{i\Phi} & \sqrt{1-\left(|r_{xx}|\cos(\Delta)\right)^{2}}e^{i\arctan(\frac{|r_{yx}|}{|r_{xx}|\sin(\Delta)})}e^{i\Phi} \\ \sqrt{1-\left(|r_{xx}|\cos(\Delta)\right)^{2}}e^{i\arctan(\frac{-|t_{yx}|}{|t_{xx}|\sin(\Delta)})}e^{i\Phi} & |r_{xx}|\text{cos}(\Delta)\cdot e^{i\Phi} \end{matrix}\right]$ (S2)

$J_{_{\text{cir}}}^{''}=\left[\begin{matrix} |r_{xx}|\text{cos}(\Delta)\cdot e^{i\Phi} & \sqrt{1-(|r_{xx}|\cos(\Delta))^{2}}e^{i\arctan(\frac{-|r_{yx}|}{|r_{xx}|\sin(\Delta)})}e^{i\Phi} \\ \sqrt{1-(|r_{xx}|\cos(\Delta))^{2}}e^{i\arctan(\frac{|r_{yx}|}{|r_{xx}|\sin(\Delta)})}e^{i\Phi} & |r_{xxx}|\text{cos}(\Delta)\cdot e^{i\Phi} \end{matrix}\right]$ (S3)

The relationship between phase of $\langle\sigma_{ll}|$, $\langle\sigma_{rr}|$, $\langle\sigma_{lr}|$, and $\langle\sigma_{rl}|$ can be established based on unitarity, symmetry, and lossless features, where, and . Without loss of generality, it can be noticed that the phase in co-polarized components is half of sum of those in two cross-polarized components, .

Building upon the seminal work^1-3^, the spin-redirection Berry phase and Pancharatnam-Berry (PB) phase can be unified under the framework of Coriolis-effect-induced phase shifts in noninertial reference frame. For spin redirection Berry phase, the critical innovation lies in implementing chiral meta-atoms to achieve spin-decoupled phase shifts through parameter-controlled cyclic evolution. Judicious modification of chiral structural parameters (e.g., *α* in Fig. 1a) enables selective Δ*φ_RL_* ∝ manipulation for LCP wave incidence while maintaining Δ*φ_LR_*≈ 0 for RCP wave incidence. Then we can conclude and Eq. S3 can be simplified as follows:

(S4)

It is important to note that the above Jones matrix ***J****_AAL_* is just a specific case of AA phase. This derivation process is also applicable to case where the AA phase is accumulated in $\langle\sigma_{lr}|$ (Eq. S5), or in both $\langle\sigma_{lr}|$ and $\langle\sigma_{rl}|$ (Eq. S6), which can further expand the number of DOFs for EM manipulation.

(S5)

(S6)

A metal-insulator-metal (MIM) configuration is employed, comprising symmetrically paired evolution arcs and circular disks interconnected via a central metallic bridge. Spin-dependent surface currents flow along the left arm-handle path under LCP wave illumination, forming coupled magnetic dipoles at the left arc-disk junction (see current distributions in Fig. S1). The arc angle *α* is modulated alter current trajectories, inducing Coriolis-effect-driven phase shifts through SO(3) polarization evolution.





1. . The surface current distributions under LCP and RCP wave illumination for three distinct arc configurations (*α* = 60°, 90°, and 120°).

Parameter-dependent scattering characteristics were systematically analyzed through full-wave electromagnetic simulations (Table S1). At 10 GHz, progressive *α* variations (40° → 140°, 10° steps) revealed distinct phase manipulation behaviors: phase in $\langle\sigma_{rl}|$ exhibited 30.8° average phase gradient versus 0.6° for counterparts in $\langle\sigma_{lr}|$. Concurrently, co-pol. scattering coefficients (LL/RR) demonstrated intermediate phase gradients of 15.6°, quantitatively confirming the spin-decoupled phase control enabled by our asymmetric meta-atom geometry.

Table S1 Numerical calculated scattering coefficients (magnitude and phase) for different *α* at *f*=10 GHz.

| *α*(deg) | 40 | 50 | 60 | 70 | 80 | 90 | 100 | 110 | 120 | 130 | 140 |
| --- | --- | --- | --- | --- | --- | --- | --- | --- | --- | --- | --- |
| *ϕ_LL_*(deg) | 16.8 | 3.6 | -10.8 | -26.4 | -44.7 | -64.2 | -82.4 | -100.1 | -114.9 | -127.8 | -139.3 |
| *ϕ_RL_*(deg) | 94.4 | 70.1 | 43.1 | 12.3 | -25.0 | -67.4 | -106.7 | -141.1 | -169.1 | -193.1 | -214.4 |
| *ϕ_LR_*(deg) | 121.3 | 117.6 | 115.3 | 114.8 | 116.0 | 117.7 | 121.4 | 120.2 | 118.5 | 116.6 | 115.0 |
| *ϕ_RR_*(deg) | 16.8 | 3.6 | -10.8 | -26.4 | -44.7 | -64.2 | -82.4 | -100.1 | -114.9 | -127.8 | -139.3 |
| |*r_LL_*| | 0.08 | 0.25 | 0.38 | 0.47 | 0.52 | 0.50 | 0.47 | 0.38 | 0.30 | 0.23 | 0.22 |
| |*r_RL_*| | 0.99 | 0.97 | 0.92 | 0.88 | 0.85 | 0.86 | 0.88 | 0.92 | 0.95 | 0.97 | 0.97 |
| |*r_LR_*| | 0.99 | 0.97 | 0.92 | 0.88 | 0.85 | 0.86 | 0.88 | 0.92 | 0.95 | 0.97 | 0.97 |
| |*r_RR_*| | 0.08 | 0.25 | 0.38 | 0.47 | 0.52 | 0.50 | 0.47 | 0.38 | 0.30 | 0.23 | 0.22 |

In the design, a low-loss dielectric substrate was selected to minimize absorption. As demonstrated in Fig. S2a, the amplitude of the emitted wave, defined as $\left| A \right|=\sqrt{\left| r_{rl} \right|^{2}+{|r_{ll}|}^{2}}$, remains at a high level ($\left| A \right|$≈1) across a broad band, except at the resonance point (*f* = 10.2 GHz), and the phase relationships between different components in the Jones matrix satisfy the relationship: Δ*φ* = *φ_xx_* + *φ_yy_* - *2φ_xy_* ± π = 0 (Fig. S2b). This confirms that the metasurface satisfies the losslessness and unitarity conditions assumed in our theoretical derivations. Furthermore, tolerance analysis of key geometric parameters demonstrates strong resilience to fabrication variations. Especially, variations in the critical parameter *r_2_* within a realistic fabrication precision range of 3.1 ± 0.02 mm result in reflection coefficient changes remain below 0.08, and phase variations are confined within ±8° across 9-14 GHz (Fig. S3), demonstrating that the meta-atom performance is highly robust to fabrication errors.





1. . (a) Amplitude of the emitted wave, (b) Phase difference Δ*φ* between cross- and co- polarized components.





1. . (a) Amplitude and (b) phase deviations introduced by fabrication errors of ±0.02 mm at parameter *r*_2_ = 3.1mm in *r_rl_* component.

## **Section S2. Jones matrix for AA and PB spin-decoupled metasurface**

To further expand the number of DOFs of Jones matrix *J_AAL_*, here, PB phase phase engineered in dual circularly polarized eigenstates. As orientation angle *θ* increases from 0° to 160°, reflection coefficients of meta-atom with *α* = 46.6° in $\langle\sigma_{lr}|$and $\langle\sigma_{rl}|$ remain constant with |*r_rl_*| *≈* 1 across 9 ~ 13 GHz (Fig. S4). And the accumulated phases are opposite for $\langle\sigma_{lr}|$and $\langle\sigma_{rl}|$ (*ϕ_rl_* = -*ϕ_lr_* = 2*θ*), as shown in Fig. S4b and S1c, while there is no phase accumulated in two co-polarized components (*ϕ_ll_* =*ϕ_rr_* =0), as shown in Fig. S4e and 1f. Resonances near 9.5 GHz and 11.8 GHz lead to an abrupt phase change in $\langle\sigma_{ll}|$, but these do not affect phase at other frequencies. The above conclusion also applies to the meta-atom with α = 137.8° (Fig. S5).


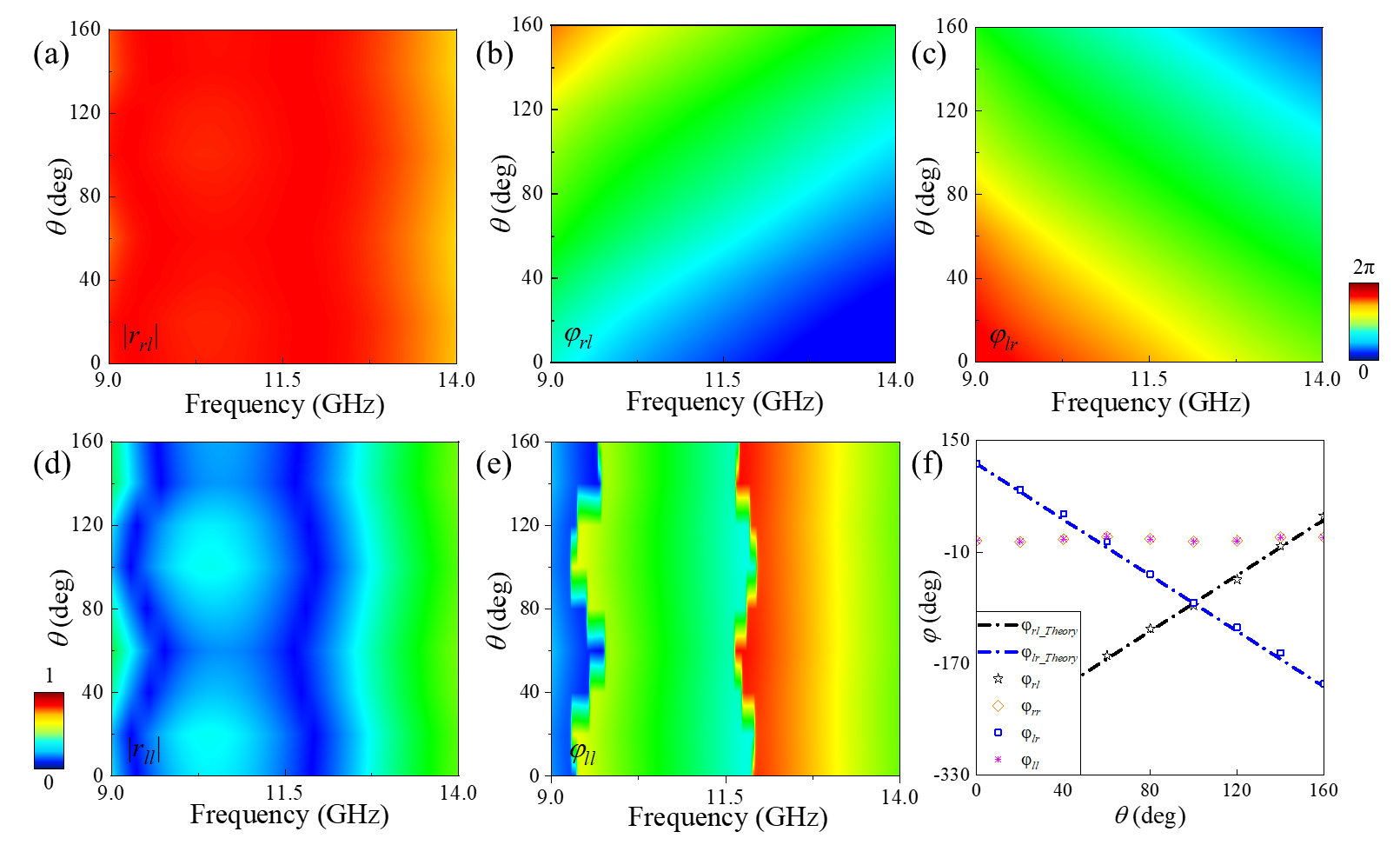


1. . Numerically calculated reflection magnitude and phase response upon rotation of the meta-atom with *α* = 46.6°. Reflection magnitude and phase response of (a-b) $\langle\sigma_{rl}|$, (c) $\langle\sigma_{lr}|$, and (d−e) $\langle\sigma_{ll}|$ at different *θ*. (f) Relationship between *θ* and phase of () and ().


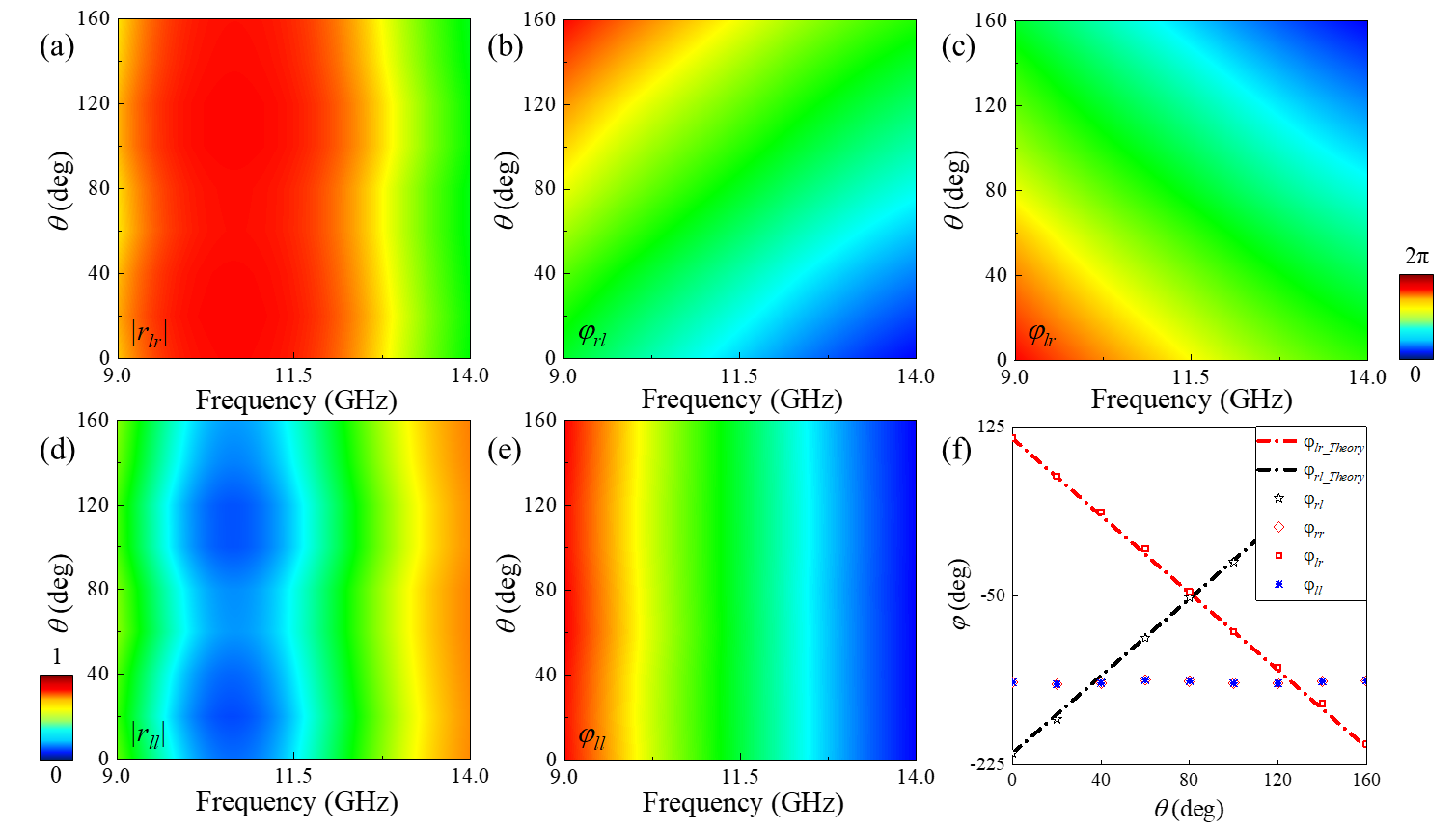


1. . Numerically calculated reflection magnitude and phase response upon rotation of the meta-atom with *α* = 137.8°. Reflection magnitude and phase response of (a-b) $\langle\sigma_{rl}|$, (c) $\langle\sigma_{lr}|$, and (d−e) at different *θ*. (f) Relationship between *θ* and phase of () and ().

Both PB and AA phase *ϕ_AAL_* can be introduced based on Eq. S6 by simultaneously changing *θ* and *α*. Thus, we can derive a more generalized form of Jones matrix with combining PB phase and AA phase as follows:

(S7)

where |*r_ll_*| = |*r_rr_*| and |*r_rl_*| = |*r_lr_*| due to planar symmetry and lossless features. Reflection magnitude of $\langle\sigma_{ll}|$ ($\langle\sigma_{rr}|$) and $\langle\sigma_{rl}|$ ($\langle\sigma_{lr}|$) are interconnected through trigonometric relationship $r_{ll}=\sqrt{1-r_{rl}^{2}}$. To maximize cross-polarization conversion efficiency, geometric structures are typically optimized to achieve |*r_rl_*| = |*r_lr_*| ≈ 1, and thus Eq. S7 can be simplified to. Phases of $\langle\sigma_{lr}|$ and $\langle\sigma_{rl}|$ can be further decoupled to compose LP wave with arbitrary polarization angle, achieving arbitrary LP wave conversion and wavefront control, as described in section 2.2 of the main text.

To validate the broadband performance of our hybrid phase framework, a focusing metasurface was engineered leveraging the *y* to 45° polarization conversion characteristics demonstrated in Fig. S6. Under y-polarized wave illumination, the metasurface achieves 45° linear polarization focusing at a designated focal frequency of 11.5 GHz with focal plane positioned at z = 205 mm. Systematic characterization reveals frequency-dependent axial focal displacement while maintaining exceptional focal quality across the 9-14 GHz (Fig. S6), conclusively demonstrating spin-decoupled phase control over a 43.5% fractional bandwidth.





1. . Performances of the designed focusing metasurface at 9 - 14 GHz under *y*-LP plane wave illuminations. Simulated electric field distributions |*E*_45°_| of 45° LP wave in *xoz* plane at (a) 9 GHz, (b) 11.5 GHz, and (c) 14 GHz. Electric field intensity |*E*_45°_| distributions at focal planes: (d) z = 162 mm, (e) z = 205 mm, (f) z = 239 mm in the *xoy* plane. (g-i) cross-sectional profiles at y = 0 mm focal positions.

**Section S3. Jones matrix and EM response for diatomic spin-decoupled metasurface with amplitude-phase controlling**

To further realize amplitude control of $\langle\sigma_{rl}|$ ($\langle\sigma_{lr}|$), diatomic meta-atom was employed based on far field interference effect of spin-decoupled meta-atom with different *θ_1_* and *θ_2_*, and then it will be represented as a summation of two Jones matrix $J_{PB+AAL_{1}}$ and $J_{PB+AAL_{2}}$, as shown in Eq. S8:

(S8)

where *θ*_1_ and *θ*_2_ represent orientation angles of meta-atoms I and II, respectively, with counterclockwise rotation denoted as positive. The Eq. S18 demonstrate that quadrature-polarized reflection coefficients can be manipulated by controlling Δ*θ* (Δ*θ* = θ_2_ -θ_1_). Here, θ_1_ is set to 0, and θ_2_ continuously increases. Fig. S7 illustrates numerically calculated reflection magnitude and phase responses of the units with *α* = 46.6° at different Δ*θ*, which is in good agreement with the findings of Eq. S8. In the diatomic metasurface governed by Eq. (S8), the amplitude-phase response reveals coupling between adjacent meta-atoms, which excites higher-order resonances near 9.5 GHz and 12.1 GHz (Fig. S7). Nevertheless, the focus of this work lies in the non-coupled frequency band, particularly near the central frequency of 10 GHz, where both amplitude and phase remain smooth with negligible coupling effects, thus ensuring stable performance across this spectral region.


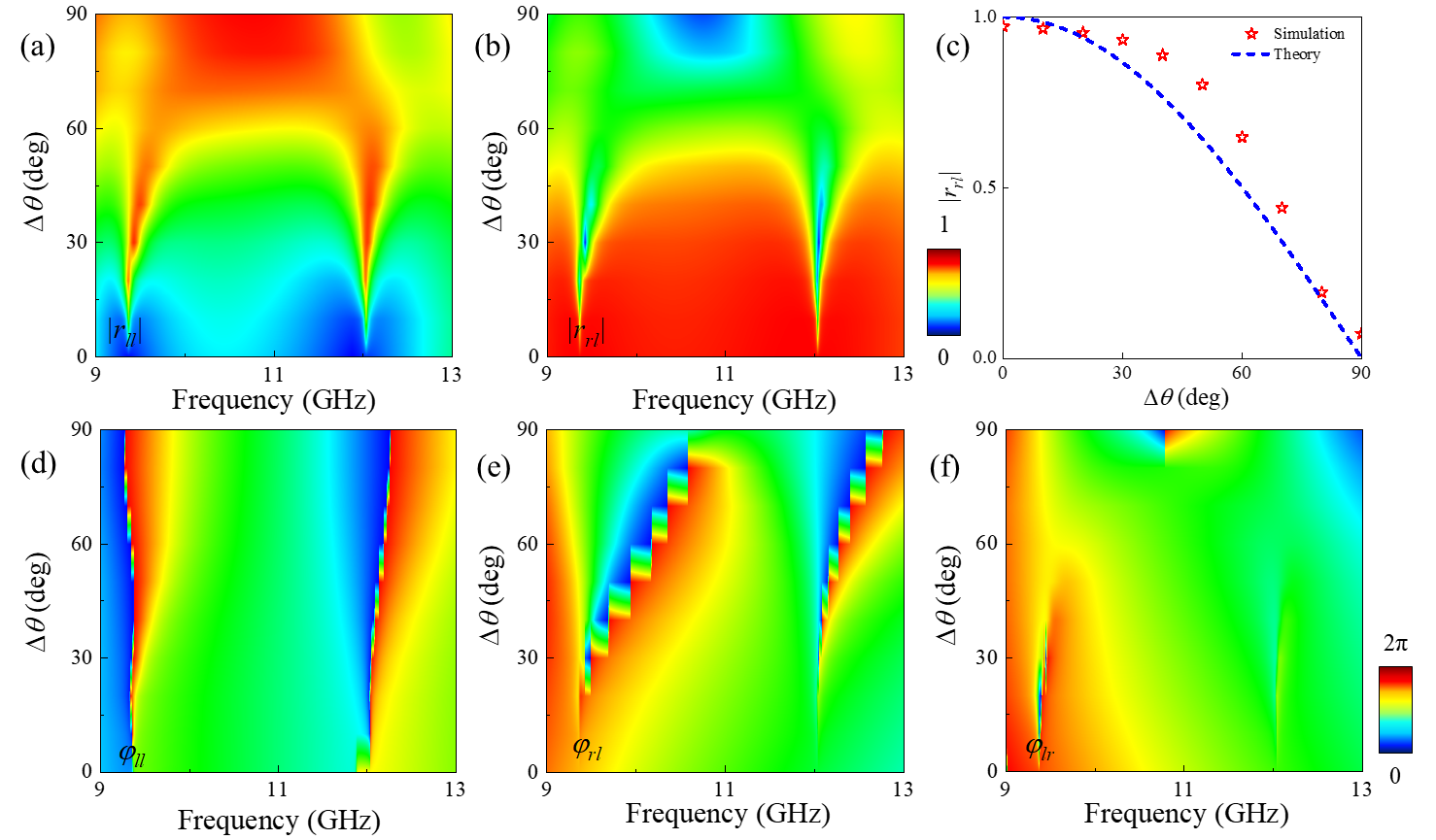


1. . Numerically calculated (a-b) reflection magnitude and (d-f) phase response of different Δ*θ* meta-atom with *α* = 46.6°. (c) Simulated and theoretical amplitudes response of the $\langle\sigma_{rl}|$ as a function of Δ*θ* at *f* =10.7 GHz.

## **Section S4. Simulated model and results of multifocal meta-device**

To maximize resolution, we proposed a novel diagonal arrangement method of meta-atoms for the design of meta-device (Fig. 4a). Real periodicity *p_r_* can be transformed from original periodicity *p* by rotating it 45° and enlarging it by $\sqrt{2}$ times (Fig. S8). Figs. S9 and S10 display measured two dimensional (2D) electric field distributions in the *yoz* plane from 10 to 12 GHz, exhibiting clear dual-focus characteristics. The relatively narrow bandwidth compared to Fig. 3 is primarily attributed to periodicity expansion and coupling between adjacent meta-atoms. Compared to periodicity *p*, the real periodicity *p_r_* of units in this arrangement expanded by a factor of $\sqrt{2}$, resulting in the phase response, amplitude response, and polarization angle 𝜓 shift towards higher frequencies (Fig. S11). Moreover, the enlarged spacing introduces coupling between adjacent meta-atoms, exacerbating phase dispersion - a collective phenomenon arising from multi-parameter interactions within $\sqrt{2}$*p*-periodic configurations (Fig. S12). This configuration induces phase difference from ideal isolated unit, resulting in focal quality variations of meta-device.


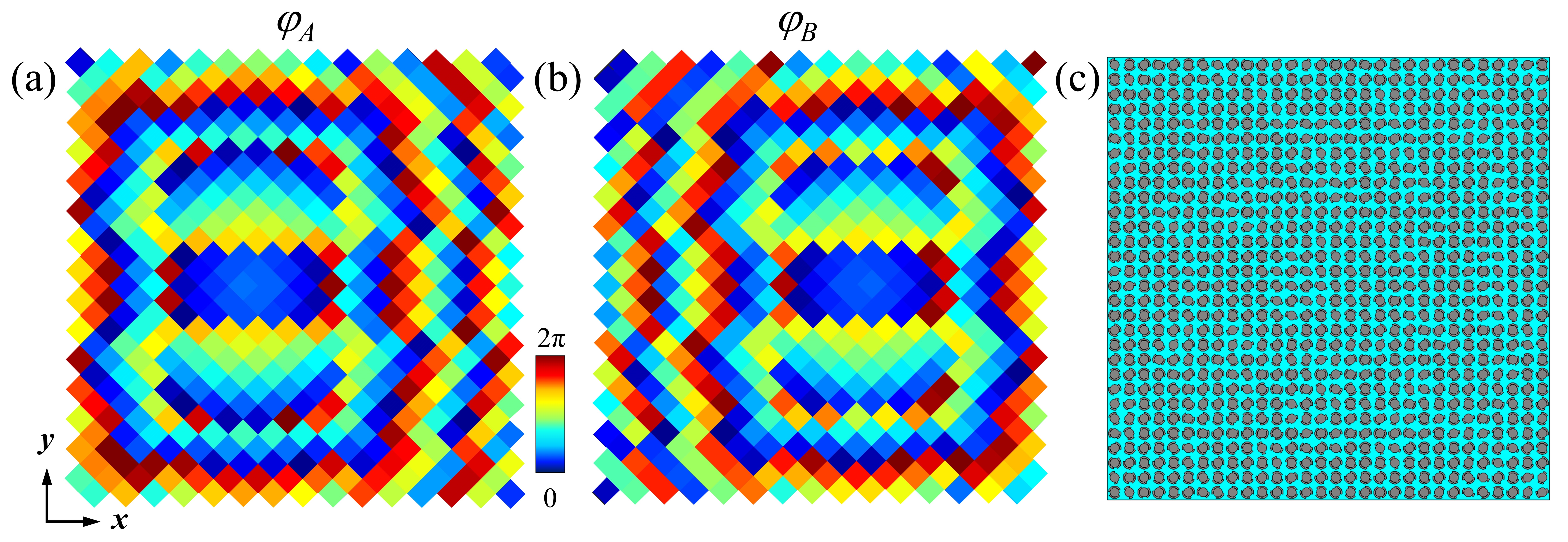


1. . Phase distributions of (a) meta-atoms A and (b) meta-atom B. (c) Simulated model of multifocal meta-device at arbitrary LP state.





1. . Measured 2D electric field distributions of -45°-LP waves in *yoz* plane for (a-c) *x* = 0 mm and (d-f) *x* = -53 mm at *f* = 10 - 12 GHz.





1. . Measured 2D electric field distributions of 45°-LP waves in *yoz* plane for (a-c) *x* = 0 mm and (d-f) *x* = 50 mm at *f* = 10 - 12 GHz.


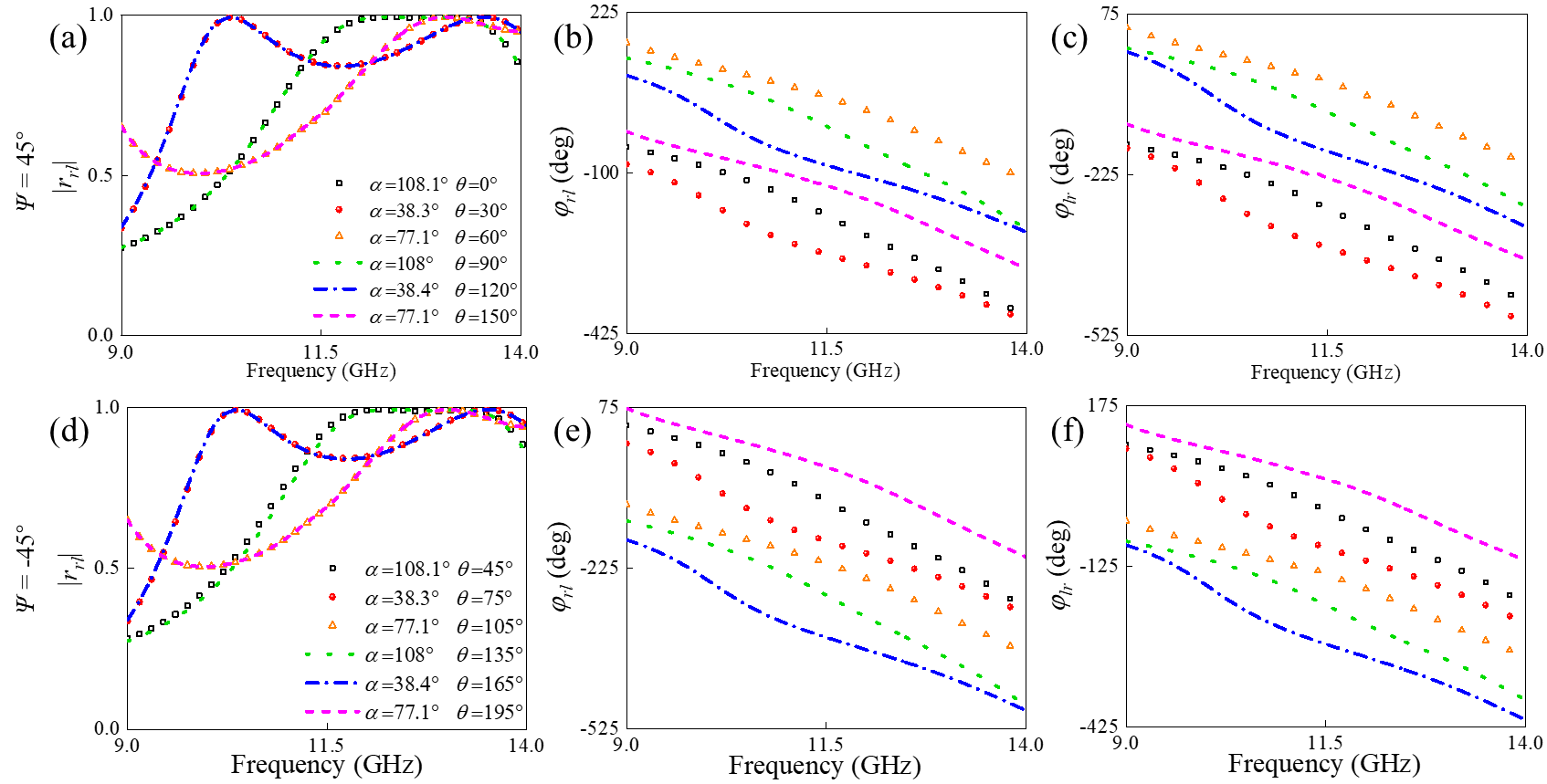


1. . Numerically calculated reflection magnitude and phase response of $\langle\sigma_{rl}|$ and $\langle\sigma_{lr}|$ of (a−c) meta-atom A and (d−f) meta-atom B at real periodicity *p_r_*.





1. . Numerically calculated phase response (a) *φ_LR_* and (b) *φ_RL_* of different meta-atoms at real periodicity *p_r_* with constant geometric parameter (*θ* = 8°, *α* = 116°) of neighboring meta-atom. (c) Frequency-dependent phase shifts of the meta-atoms at *f* = 11.8 GHz.

## **Section S5. Simulated model and experimental setups of diatomic meta-device with beam deflection**

Under oblique incidence conditions, the meta-atoms exhibit frequency-dependent amplitude-phase characteristics. For $\langle\sigma_{lr}|$, meta-atom I shows a transmission loss of 1.5 dB and phase variation of 28° at 30° incidence angle, and meta-atom II demonstrates minimal loss and 40° phase variation (Fig. S13). The observed loss originates from the fact that the resonance frequency of metaatom-I at 9.96 GHz shifts to higher one with increasing incidence angle, which is close to the operating frequency. The amplitude in $\langle\sigma_{rr}|$ can be calculated based on lossless feature, and the phase is consistent with that of $\langle\sigma_{lr}|$. Notably, edge-positioned meta-atoms of the array only operate at high incidence angles, resulting in slight perturbations to the overall EM characteristics.

The experimental setup employed a CP transmitter, aligned at (*x*,*y*,*z*) = (0, 0, 205 mm) relative to the array plane. Then, the beam-deflected phase profile of the array was calculated based on the source position with reconstructed phase distribution and simulation model of arry (Fig. S14 a and b). To validate the derived phase-amplitude relationship in co-polarized components, we designed and simulated a polarization-preserving metasurface for 25° beam deflection. The metasurface exhibits consistent co-polarized reflection under arbitrary incident polarizations with a meta-atom orientation difference of *θ*₁ - *θ*₂ = 90°. Simulated result under RCP and elliptical polarized (*χ* = 18.9°, *ψ* = 45°) wave incidence confirm that the polarization state remains unchanged, while simultaneously imparting the desired wavefront phase profile (Fig. S15), verifying our amplitude-phase control strategy. Experimental validation of scattering pattern was performed using experiment setup (Fig. S16).





1. . Numerically calculated amplitude and phase responses of 1-bit meta-atoms in (a-b) co- and (c-d) cross-polarized scattering coefficient under oblique incidence conditions.





1. . (a) Phase distribution and (b) simulated model of diatomic meta-device for beam deflection, where the *F* and *D* signify focal length and aperture dimension, respectively.





1. . 1D far-field scattering patterns of the metasurface (with rotation angles *θ*₁ = 0° and *θ*₂ = 90°) under RCP and elliptically polarized waves illumination.





1. . Schematic diagram of experiment setups for (a) near-field and (b) far-field measurements.

To emphasize the innovative advantages of our research, we compared previous chiral metasurface with our proposed AA and PB phase strategy (as summarized in Table S2 and S3). The analysis reveals that previous methods, constrained by structural chirality or intrinsic material chirality, operate exclusively under specific CP conditions. For single-phase designs (e.g., solely AA or PB phase), limited degrees of freedom restrict phase control for broadband polarization angle tuning (*χ_out_* = –*χ_in_*, *ψ_out_* ≠-*ψ_in_*) of arbitrary polarized waves. Our AA-PB synergistic approach provides additional degrees of freedom achieving arbitrary linear-to-linear polarization conversion with wavefront control (9-14 GHz). Furthermore, integrated amplitude modulation further expands the range of accessible SU(2) operations, allowing circular-to-arbitrary polarization conversion with wavefront manipulation, as demonstrated in Table S2.

Table S2 Performance comparison of polarization and phase manipulation via AA phase, PB phase, and their synergy.

| Single DoF | PB phase  (polarization conversion) | $\left[ \begin{matrix} 0 & e^{i2\theta} \\ e^{-i2\theta} & 0 \end{matrix} \right]$ |
| --- | --- | --- |
|  | AA phase  (polarization conversion) | $\left[ \begin{matrix} 0 & 1 \\ e^{i\varphi_{AA}} & 0 \end{matrix} \right]$ |
| Double DoFs | AA and PB phase  (polarization conversion and phase manipulation) | $\left[ \begin{matrix} r_{ll}e^{i\frac{\varphi_{AA}}{2}} & r_{lr}e^{i2\theta} \\ r_{rl}e^{i\varphi_{AA}-i2\theta} & r_{rr}e^{i\frac{\varphi_{AA}}{2}} \end{matrix} \right]$ |
| Triple DoFs | AA and PB phase  (polarization conversion and phase manipulation) |  |

Table S3 Comparison of key parameters of chiral metasurfaces.

| Ref | Frequency band (fractional bandwidth) | Polarization Conversion Efficiency of chiral meta-atom | Polarization states of emitted wave | Transmission /Reflection | Amplitude/  Phase |
| --- | --- | --- | --- | --- | --- |
| [4] | 14GHz | ≈40% | CP | Transmission | Amplitude |
| [5] | 700-850 nm (19.4%) | ≈8% | CP | Transmission | Phase |
| [6] | 980-1200 nm (20.18%) | ≈68% | CP | Reflection | Phase |
| [7] | 1.245-1.55 μm (21.8%) | ≈56%(reaching up to 74%) | CP | Reflection | Phase |
| [8] | 915 nm | ranging from 72% to 97% | Arbitrary linear polarization | transmission | Phase |
| Our work | 9-14 GH (43.5%) | ＞75% (reaching up to 99.5%) | Arbitrary linear polarization | Reflection | Phase |
|  | 10 GHz | reaching up to 99% | Arbitrary polarization | Reflection | Phase |

**Reference**

1. G.D. Bai, Q. Ma, R.Q. Li, J. Mu, H.B. Jing, L. Zhang, T.J. Cui, "Spin-Symmetry Breaking Through Metasurface Geometric Phases", *Physical Review Applied*. vol. 12, no. 4, 2019, pp. 044042.
2. R. Ji, X. Xie, X. Guo, Y. Zhao, C. Jin, K. Song, S. Wang, J. Yin, Y. Liu, C. Jiang, C. Yang, X. Zhao, W. Lu, "Chirality-Assisted Aharonov–Anandan Geometric-Phase Metasurfaces for Spin-Decoupled Phase Modulation", *ACS Photonics*. vol. 8, no. 6, 2021, pp. 1847-1855.
3. K.Y. Bliokh, Y. Gorodetski, V. Kleiner, E. Hasman, "Coriolis Effect in Optics: Unified Geometric Phase and Spin-Hall Effect", *Physical Review Letters*. vol. 101, no. 3, 2008, pp. 030404.
4. V.A. Fedotov, P.L. Mladyonov, S.L. Prosvirnin, A.V. Rogacheva, Y. Chen, N.I. Zheludev, "Asymmetric Propagation of Electromagnetic Waves through a Planar Chiral Structure", *Physical Review Letters*. vol. 97, no. 16, 2006, pp. 167401.
5. Y. Chen, X. Yang, J. Gao, "3D Janus plasmonic helical nanoapertures for polarization-encrypted data storage", *Light: Science & Applications*. vol. 8, no. 1, 2019, pp. 45.
6. C. Chen, S. Gao, W. Song, H. Li, S.-N. Zhu, T. Li, "Metasurfaces with Planar Chiral Meta-Atoms for Spin Light Manipulation", *Nano Letters*. vol. 21, no. 4, 2021, pp. 1815-1821.
7. R. Jin, L. Deng, L. Tang, Y. Cao, Y. Liu, Z.-G. Dong, "Decoupled Phase Modulation for Circularly Polarized Light via Chiral Metasurfaces", *ACS Photonics*. vol. 10, no. 1, 2023, pp. 155-161.
8. A. Arbabi, Y. Horie, M. Bagheri, A. Faraon, "Dielectric metasurfaces for complete control of phase and polarization with subwavelength spatial resolution and high transmission", *Nature Nanotechnology*. vol. 10, no. 11, 2015, pp. 937-943.
